# Protocols for anticoagulation management in pediatric extracorporeal membrane oxygenation: A comparative retrospective study

**DOI:** 10.1177/02676591261416084

**Published:** 2026-01-21

**Authors:** Mirte L. Jansen, Nina C. Boom, Nienke Schalij, Peter Paul Roeleveld, Eelco Van Es, Oliver Karam, Ariane Willems

**Affiliations:** 1 Pediatric Intensive Care Unit, Department of Intensive Care, Leiden University Hospital, Leiden, The Netherlands; 2 Department of Pediatrics, Leiden University Hospital, Leiden, The Netherlands; 3 Department of Cardiothoracic Surgery, Leiden University Hospital, Leiden, The Netherlands; 4 Pediatric Critical Care Medicine, Department of Pediatrics, 12228 Yale School of Medicine, New Haven, CT, USA; 5 Pediatric Intensive Care Unit, Department of Pediatrics, University Hospital Brussels. University Children’s Hospital Queen Fabiola, Brussels, Belgium

**Keywords:** pediatrics, anticoagulation, extracorporeal membrane oxygenation, antifactor Xa, activated partial thromboplastin time, activated clotting time, heparin, hemorrhage

## Abstract

**Introduction:**

In children undergoing Extracorporeal Membrane Oxygenation (ECMO), anticoagulation is given to counterbalance the risk of thrombosis. Several laboratory tests are available to monitor heparin, but the ideal method still needs to be determined.

**Methods:**

This retrospective cohort study included all patients under 18 years on ECMO support between 2010 and 2021. At our institution, the test used to monitor unfractionated heparin changed over time, dividing patients into three periods, using either activated clotting time (ACT) (2010-2014), activated partial thromboplastin time (aPTT) (2014-2018) or anti-Xa (2018-2021). The primary objective was to compare the occurrence of hemorrhagic complications. Secondary objectives included thrombotic complications, neurological complications, and survival.

**Results:**

We included 118 ECMO runs of which 30 ACT-guided, 40 aPTT-guided, and 48 anti-Xa-guided. No statistically significant differences were found in hemorrhagic complications [respectively 46.7% vs. 52.5% vs. 60.4%; *p* = 0.48], thrombotic complications (*p* = 0.15), neurological complications (*p* = 0.13), or 30-days survival (*p* = 0.84). Duration of ECMO and length of hospital stay were both the shortest in the anti-Xa-guided group (respectively *p* = 0.02; *p* = 0.02). During ECMO, the anti-Xa-guided group received a higher unfractionated heparin dose compared to the aPTT- and ACT-guided group [respectively 839 [651–981] vs. 543 [407–692] vs. 330 [223–489] IU/kg d^-1^, *p* < 0.001].

**Conclusion:**

In our study, the test or titration method used to guide heparin-dosing in children on ECMO, was not associated with hemorrhagic complications and death. Of note, the dose of unfractionated heparin was significantly higher in the anti-Xa-guided group. Combined testing may be more effective than a single method, more studies are needed to establish the optimal strategy.

## Introduction

Extracorporeal membrane oxygenation (ECMO) provides support for refractory respiratory and/or circulatory failure.^1^ According to the Extracorporeal Life Support Organization (ELSO) Registry, over 20,564 neonates and 25,739 children worldwide have been treated with ECMO between 2009 and 2022.^2^ ECMO exposes the blood of the patient to several artificial surfaces that activate the coagulation cascade, platelets, and induces inflammation.^3^ In children, anticoagulation is frequently considered mandatory to prevent thrombotic complications. An American survey showed that 94% of institutions use unfractionated heparin (UFH) as their primary anticoagulant in pediatric ECMO patients for its numerous advantages including low cost, short half-life, reversal with protamine, and familiarity among clinical users.^1,4,5^ UFH interacts with antithrombin (AT) and tissue factor pathway inhibitor (TFPI). It binds to the enzyme inhibitor AT, thereby enhancing the anticoagulant activity of AT on free thrombin by a 1000-fold. Given that UFH also binds to tissue factor pathway inhibitor, the inhibition of factor Xa is catalyzed and enhanced by two-to four-fold, contributing to its anticoagulant effect.^6^

Thrombotic and hemorrhagic complications are common in children receiving ECMO and represent a significant cause of morbidity and mortality.^7^ Bleeding and clotting occur in respectively 70% and 38% of pediatric ECMO patients.^8^ Therefore, achieving the right balance between thrombosis and bleeding remains a major challenge in this population.

Over the years, several methods have become available to monitor and titrate UFH, but the ideal method still needs to be determined.^9^ First, the activated clotting time (ACT), utilized since the 1970s when ECMO was first introduced. It is the method of choice used for UFH titration during cardiopulmonary bypass.^10^ The ACT represents the time in seconds necessary for whole blood to form a fibrin clot after the addition of various coagulation activators.^9^ Several factors can influence the ACT, including platelet count and function, D-dimer level, fibrinogen levels, coagulation factor deficiencies, temperature, hemodilution, and various technical factors.^9^ Because the ACT measures the end point of the clotting cascade it does not solely represent the effect of UFH. Several studies support this by showing a poor correlation of ACT to the UFH dose.^11–14^ However, it is important to note that different ACT tests exist, and that the results can vary according to the activator and/or concomitant use of hemostatic agents and fibrinolytics. An alternative method is the activated partial thromboplastin time (aPTT), a laboratory test developed for monitoring UFH therapy.^9^ It measures plasma coagulation by determining the time, in seconds, from factor XII activation to fibrin formation. It reflects the activity of both UFH and patient coagulation factors but cannot differentiate between the two. The aPTT is therefore sensitive to changed levels of coagulation factors as well as non-coagulation factors like lupus antigen.^9,15,16^ Furthermore, it has a high inter- and intra-patient variability, especially in infants, with a poor correlation to the UFH dose.^1^ Additionally, the aPTT depends on the reagent used which results in differences between tests and laboratories. Finally, the UFH can also be titrated using the anti-Xa level, which measures the anticoagulation ability of UFH to catalyze AT’s inhibition of factor Xa activity.^9^ Acute phase reactants affect both the aPTT and the ACT, whilst the anti-Xa level is only influenced by AT. Anti-Xa is therefore more representative of the UFH dose.^17^ There are two types of anti-Xa tests, an optical density based-method and a clotting-based method. The optical density method can be falsely low in presence of increased plasma free hemoglobin, triglycerides, and bilirubin.^9^ Currently, there is no consensus on which management strategy is associated with better outcomes. At our center, different anticoagulation strategies for UFH titration were used over time. The goal of our study was to compare those different strategies on hemorrhagic complications in children undergoing ECMO. Secondary objectives included thrombotic complications, neurological complications, and survival.

## Method

### Design

This single-center retrospective cohort study was performed at the Pediatric Intensive Care Unit (PICU) of the Leiden University Medical Centre and included all consecutive patients younger than 18 years of age who underwent ECMO between January 1^st^ 2010 and December 31^st^ 2021. Patients were identified using our local quality control ECMO database (Metavision suite, iMDsoft). Exclusion criteria included patients with a hereditary coagulation disorder, patients treated with a different anticoagulant than UFH or receiving alteplase during ECMO, and patients lacking data on ECMO parameters or UFH. The study protocol was approved by the Institutional Review Board (Reference number: G20.147; November 10^th^, 2020) and informed consent was waived due to the retrospective character of the study.

### Data collection

Data was collected from the patients’ electronic medical records and included demographic data, type of ECMO, site of cannulation, known underlying disorders, and indication for ECMO. The outcomes assessed included hemorrhagic, thrombotic, and neurological complications, as well as mortality, the incidence of necrotic enterocolitis (NEC), infection, and the need for renal replacement therapy (RRT). Additionally, the study evaluated the duration of ECMO and mechanical ventilatory support, as well as, length of stay in both the intensive care unit (ICU) and the hospital. The UFH dosing (expressed as IU/kg d^-1^), including boluses and infusions rates, was also extracted from the patient’s charts. Furthermore, data on the use of blood products, anti-hemostatic agents, and anti-fibrinolytic agents was collected.

### Definition of complications

The definitions of complications are presented in Supplement 1. Hemorrhagic complications were defined as the standardized definition from the ELSO Registry extended by the presence of gastro-intestinal, pulmonary, and intracranial hemorrhage. Thrombotic complications were defined as patient-related thrombus in the vascular or central nervous system, intracardiac thrombosis, limb ischemia, pulmonary embolism, and circuit or component changes due to clot formation or mechanical failure. Neurological complications were also defined according to the ELSO Registry and involved seizures, diffuse ischemia or infarction in the central nervous system, intraventricular hemorrhage and supra/intra parenchymal hemorrhage. All complications had to occur during ECMO or within 14 days after decannulation and in-hospital. In addition, infections during ICU stay, NEC, and RRT were registered.

### Extracorporeal membrane oxygenation and anticoagulation management

During the study period, all patients were supported on an oxygenator and a centrifugal pump (Revolution®, Italy) with both phosphorylcholine coating. Cannulas were not coated. In general, circuit flows ranged between 100 and 150 mL/kg/min. ECMO circuits were primed with one unit of packed red blood cells (RBCs) and approximately 150 ml of NaCl 0.9%. During the study period, no changes were made in the ECMO system components.

At initiation of ECMO, patients received a bolus UFH of 50-100 IU/kg followed by a continuous UFH infusion started at 20 IU/kg h^-1^. However, in patients following cardiac surgery, the administration of a bolus and the timing of starting UFH continuous infusion, depended on the amount of blood loss and was left at the discretion of the cardiac surgeon. In the whole cohort and along the whole study period, complete blood count, aPTT, prothrombin time, international normalized ratio and fibrinogen were drawn at least four times a day. UFH titration strategies changed over time. At first, ACTs were used for the titration of UFH. ACTs were determined every hour and kept between 160 and 180s. UFH was lowered or increased by 2-4 IU/kg h^-1^ based on the most recent laboratory result. In case of bleeding the target was adapted to an ACT of 140-160s. Twenty-four to 48 hours after the bleeding stopped target values were changed back to 160-180s. Secondly, from 2014 till 2018, the protocol was adapted to an aPTT-based strategy. The aPTT was measured four times a day and targeted 1.5–2.5 times the upper limit of normal. For non-surgical patients this ranged between 60 and 80s. The continuous infusion of UFH started at 25-30 IU/kg h^-1^. For surgical patients, the aPTT target ranges between 50 and 70s. The UFH infusion started at 15-20 UI/kg h^-^1. Later in 2018, the anticoagulation protocol changed to anti-Xa-guided titration. Anti-Xa was analyzed four times a day and kept between 0.3 and 0.7 IU/mL. In our center the optical density anti-Xa test was used, and no exogenous AT was added to the anti-Xa assay (STA-R Max®). In case prothrombin complex concentrates (PCC, Cofact®) or plasma were indicated the choice between the two was left at the discretion of the treating intensivist.

### Statistical analysis

The normality of continuous variables was assessed using the Kolmogorov-Smirnov test. Descriptive data were reported as mean and standard deviation (SD), or as median and interquartile ranges, depending on the data distribution. Categorical data were visualized in absolute numbers and percentages. For nonparametric continuous data, patient groups were compared using the Kruskal Wallis test. When significance was found a Mann-Whitney test with post-hoc Bonferroni correction was performed to identify the deviating group, a p-value of 0.0167 or less was considered significant. For normally distributed continuous data, the ANOVA F-test was used. When significance was found a Tukey’s HSD post-hoc test was conducted to identify the deviating group. For categorical data, the Chi-square test was used to assess the overall difference between groups, a *p*-value of 0.05 or less was considered significant. In case significance was found a post-hoc pairwise comparison with Bonferroni correction was conducted to identify the deviating group, a *p*-value of 0.0167 or less was considered significant. A Kaplan-Meier analysis was performed for the 30-day survival to decannulation, statistical significance was determined with the log-rank Mantel-Cox test. Statistical analyses were performed using IBM SPSS statistics 25.0. An additional sensitivity analysis was performed to take into account the possible bias of some protocol violations, where multiple titrations methods were used to titrate the UFH dosage.

## Results

### Primary analysis

#### Patient characteristics

From January 2010 to December 2021, 125 ECMO runs were included in the study, of which seven runs were excluded, leaving 118 ECMO runs for analysis: 30 runs in the ACT-guided group, 40 runs in the aPTT-guided group, and 48 runs in the anti-Xa-guided group (Figure 1). Baseline characteristics are listed in Table 1.

**Figure 1.**
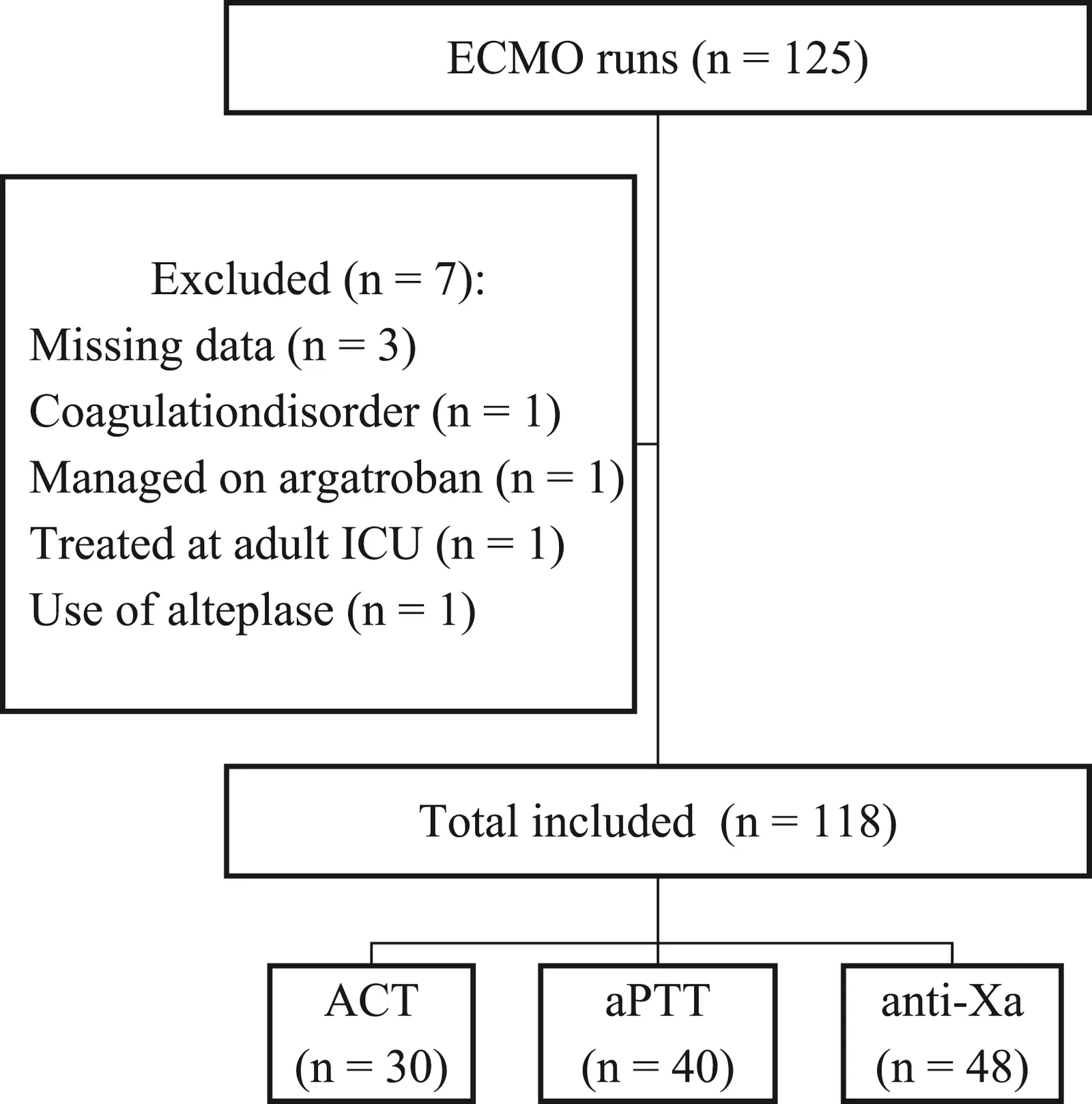
Flow diagram. ECMO, extracorporeal membrane oxygenation; ICU, intensive care unit; ACT, activated clotting time; aPTT, activated partial thromboplastin time; anti-Xa, antifactor Xa.

**Table 1.** Baseline characteristics.

	Total (n = 118)	ACT (n = 30)	aPTT (n = 40)	anti-Xa (n = 48)	*p*
Sex, males, no. (%)	52 (44.1)	13 (43.3)	16 (40.0)	23 (47.9)	*0.754*
Age (days)^a^	67.2 (9.8–290.4)	135.8 (16.3–290.4)	102.3 (17.8–328.7)	32.4 (2.3–125.8)	*0.120*
Weight (kg)^a^	4.3 (3.5–7.4)	5.2 (3.4–7.6)	4.8 (3.8–7.5)	4.0 (3.0–6.8)	*0.219*
Veno-arterial, no. (%)	114 (96.6)	30 (100)	39 (97.5)	45 (93.8)	*0.309*
Central cannulation (n = 114), no. (%)	64 (54.2)	19 (70.4)	18 (46.2)	27 (56.3)	*0.150*
Disorder, no. (%)					*0.094*
Chromosomal	14 (11.9)	7 (23.3)	3 (7.5)	4 (8.3)	
Infectious	7 (5.9)	2 (6.7)	4 (10.0)	1 (2.1)	
Congenital cardiac	94 (79.7)	27 (90.0)	36 (90.0)	31 (64.6)	
Acquired cardiac	13 (11.0)	2 (6.7)	6 (15.0)	5 (10.4)	
Pulmonary hypertension	28 (23.7)	5 (16.7)	9 (22.5)	14 (29.2)	
Congenital lung	12 (10.2)	0	3 (7.5)	9 (18.8)	
Acquired lung	8 (6.8)	2 (6.7)	4 (10.0)	2 (4.2)	
Indication ECMO, no. (%)					*0.108*
Cardiac	101 (85.6)	28 (93.3)	34 (85.0)	39 (81.3)	
Respiratory	29 (24.6)	5 (16.7)	11 (27.5)	13 (27.1)	
ECPR	37 (31.4)	6 (20.0)	13 (32.5)	18 (37.5)	
Sepsis	3 (2.5)	3 (10.0)	0	0	
Postoperative	59 (50.0)	19 (63.3)	20 (50.0)	20 (41.7)	

ACT: activated clotting time; aPTT: activated partial thromboplastin time; anti-Xa: antifactor Xa; ECMO: extracorporeal membrane oxygenator; ECPR: extracorporeal cardiopulmonary resuscitation.

^a^Median (IQR).

### Outcomes

Outcomes are presented in Table 2. The primary outcome, hemorrhagic complications, occurred in 46.7% of patients in the ACT-guided group, 52.5% in the aPTT-guided group, and 60.4% in the anti-Xa-guided group, with no statistical significant difference between the groups (*p* = 0.477). Thrombotic complications occurred in 50.0% of the patients in the ACT-guided group, 52.5% of the aPTT-guided group, and 33.3% of the anti-Xa-guided group (*p* = 0.148). Neurological complications were noticed in 20.0% of the ACT-guided group, 37.5% of the aPTT-guided group, and 41.7% of the anti-Xa-guided group (*p* = 0.134). No difference was seen for other complications. The duration of ECMO and length of hospital stay differed significantly across the three groups (respectively *p* = 0.022; *p* = 0.020), the post-hoc analysis identified significance between anti-Xa-guided and aPTT-guided titration. Both duration of intubation and length of ICU stay were similar over all groups (respectively *p* = 0.053; *p* = 0.066). The log-rank test revealed no statistically significant difference in survival 30-days after decannulation (*p* = 0.836) (Figure 2).

**Table 2.** Clinical course and complications.

	ACT (n = 30)	aPTT (n = 40)	anti-Xa (n = 48)	*p*
Duration of ECMO (days)^a^	5.5 (3.4–7.6)^b^	5.9 (3.2–8.7)^b^	3.5 (2.0–5.8)^b^	*0.022*
Duration of intubation (days)^a^	20.9 (11.0–26.4)	26.8 (11.9–38.6)	17.2 (7.9–28.1)	*0.053*
Hospital LOS (days)^a^	38.5 (22.0–83.0)^b^	56.0 (29.3–98.8)^b^	31.5 (15.0–58.0)^b^	*0.020*
ICU LOS (days)^a^	26.0 (13.8–54.5)	44.5 (19.8–64.5)	21.0 (8.3–52.0)	*0.066*
Mortality on ECMO, no (%)	6 (20.0)	12 (30.0)	15 (31.3)	*0.526*
Hemorrhagic, no. (%)	14 (46.7)	21 (52.5)	29 (60.4)	*0.477*
Surgical site	9 (30.0)	9 (22.5)	17 (35.4)	*0.417*
Cannula-site	4 (13.3)	13 (32.5)	11 (22.9)	*0.173*
Gastro-intestinal	1 (3.3)	1 (2.5)	0	*0.480*
Intracranial	1 (3.3)	3 (7.5)	7 (14.6)	*0.223*
Pulmonary	1 (3.3)	6 (15.0)	2 (4.2)	*0.096*
Thrombotic, no. (%)	15 (50.0)	21 (52.5)	16 (33.3)	*0.148*
Patient thrombus	6 (20.0)	10 (25.0)	7 (14.6)	*0.469*
Circuit change	3 (10.0)	11 (27.5)	5 (10.4)	*0.054*
Component change	8 (26.7)	7 (17.5)	4 (8.3)	*0.096*
Intracardiac	2 (6.7)	3 (7.5)	2 (4.2)	*0.789*
Limb ischemia	1 (3.3)	2 (5.0)	0	*0.316*
Pulmonary embolism	1 (3.3)	0	1 (2.1)	*0.544*
Neurological, no. (%)	6 (20.0)	15 (37.5)	20 (41.7)	*0.134*
Seizure	2 (6.7)	7 (17.5)	8 (16.7)	*0.374*
CNS Diffuse ischemia	1 (3.3)	3 (7.5)	6 (12.5)	*0.354*
CNS Infarction	3 (10.0)	7 (17.5)	7 (14.6)	*0.676*
Intraventricular CNS hemorrhage	0	1 (2.5)	3 (6.3)	*0.309*
Intra/extra parenchymal CNS hemorrhage	1 (3.3)	2 (5.0)	5 (10.4)	*0.413*
Infection, no. (%)	11 (36.7)	15 (37.5)	11 (22.9)	*0.261*
NEC, no. (%)	2 (6.7)	3 (7.5)	8 (16.7)	*0.266*
RRT, no. (%)	5 (16.7)	12 (30.0)	13 (27.1)	*0.422*

ACT: activated clotting time; aPTT: activated partial thromboplastin time; anti-Xa: anti-factor-Xa; ECMO: extracorporeal membrane oxygenator; LOS: length of stay; ICU: intensive care unit; CNS: central nervous system; NEC: necrotic enterocolitis; RRT: renal replacement therapy.

^a^Median (IQR).

^b^Kruskal Wallis tests were used to compare between various groups. For post-hoc testing pairwise Mann Whitney tests, with the Bonferroni correction (*p* < 0.0167), were performed.

**Figure 2.**
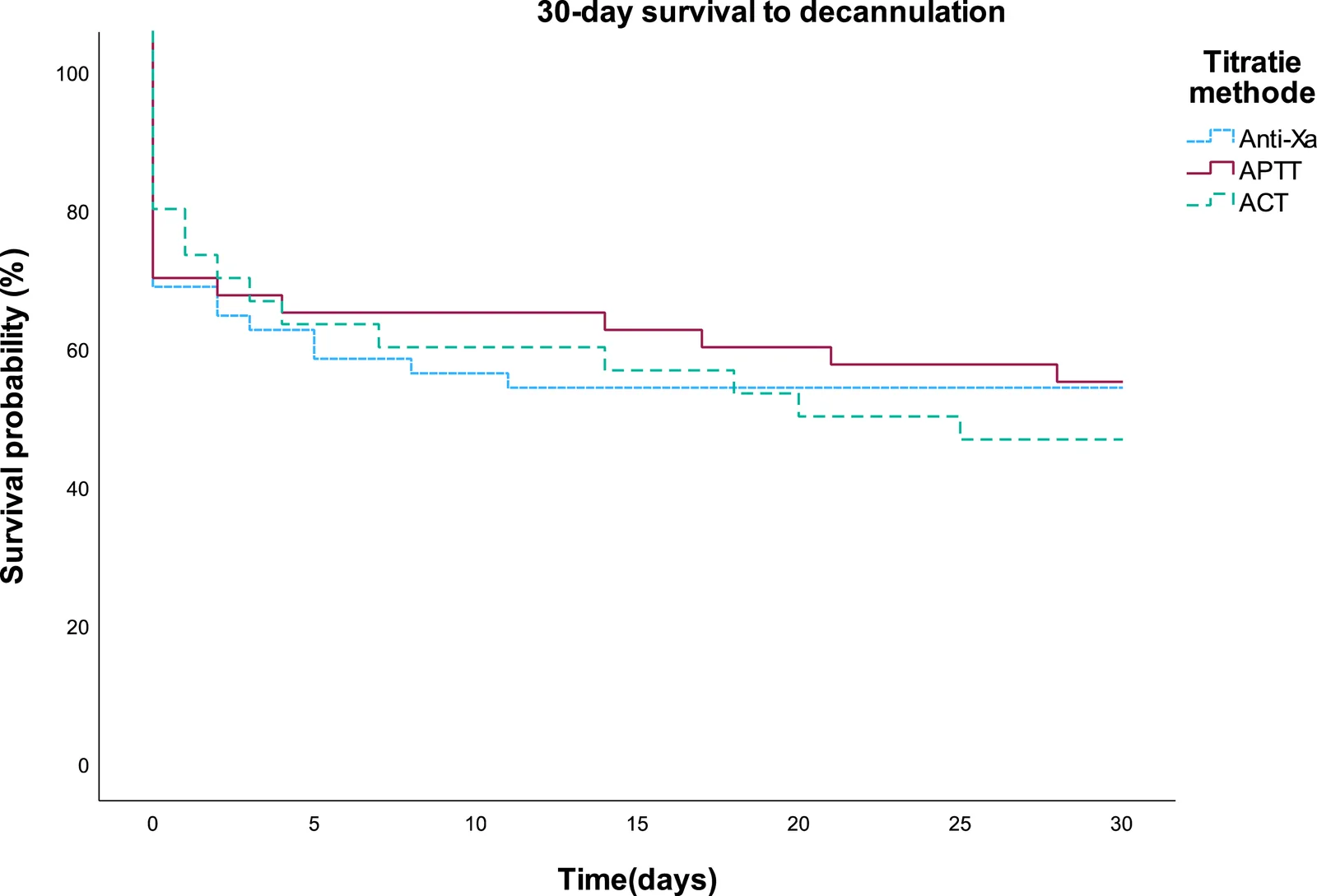
Kaplan-Meier analysis of 30-days survival after decannulation from extracorporeal membrane oxygenation support. Log rank test (*p* = 0.836).

### Anticoagulation and exposure to blood products

Table 3 presents the UFH dose given. The anti-Xa-guided group received significantly higher UFH doses than both the aPTT-guided and the ACT-guided group [838.6 IU/kg d^-1^, 543.4 IU/kg d^-1^ and 329.9 IU/kg d^-1^ respectively; *p* < 0.001]. An UFH bolus was given to 86.7% of the patients in the ACT-guided group, 62.5% of the aPTT-guided group, and 87.5% of the anti-Xa-guided group (*p* = 0.008), post-hoc analysis identified significance between aPTT- and anti-Xa-guided titration. In the aPTT-guided group the dosage of the bolus given was lowest (ACT 300 IU/kg vs aPTT 100.0 IU/kg vs anti-Xa 142.7 IU/kg, *p* = 0.006). The continuous heparin infusion was started after 10.5 [2.5 – 13.9] hours in the ACT-guided group, 3.7 [1.6 – 54.4] hours in the aPTT-guided group and 3.0 [1.9 – 5.8] hours in the anti-Xa-guided group (*p* = 0.020), post-hoc analysis identified significance between ACT- and anti-Xa-guided titration.

**Table 3.** Unfractionated heparin dosing.

	ACT (n = 30)	aPTT (n = 40)	anti-Xa (n = 48)	*p*
Bolus, no. (%)	26 (86.7)^c^	25 (62.5)^c^	42 (87.5)^c^	*0.008*
Bolus (IU/kg) (n = 93)^a^	300.0 (108.3–625.4)^d^	100.0 (46.3–166.5)^d^	142.7 (75.0–189.6)^d^	*0.006*
Timing continuous UFH				*0.611*
Before start ECMO, no. (%)	2 (6.7)	1 (2.5)	3 (6.3)	
Simultaneously, no. (%)	4 (13.3)	7 (17.5)	12 (25.0)	
After start ECMO, no. (%)	24 (80.0)	32 (80.0)	33 (68.8)	
Delay (hours)^a^	10.5 (2.5–13.9)^d^	3.7 (1.6–5.4)^d^	3.0 (1.9–5.8)^d^	*0.020*
Total dosage (IU/kg d^-1^)^a^	329.9 (223.3–488.9)^d^	543.4 (407.3–691.9)^d^	838.6 (651.0–981.1)^d^	*<0.001*
Mean rate (IU/kg h^-1^)^b^	12.5 (10.2)^e^	21.8 (8.1)^e^	31.5 (10.1)^e^	*<0.001*
Minimal rate (IU/kg h^-1^)^a^	5.0 (3.0–9.5)^d^	10.5 (7.6–20.0)^d^	23.4 (20.0–27.5)^d^	*<0.001*
Maximal rate (IU/kg h^-1^)^a^	24.5 (16.7–30.0)^d^	30.3 (25.0–39.5)^d^	41.0 (34.2–48.1)^d^	*<0.001*

ACT: activated clotting time; aPTT: activated partial thromboplas**t**in time; anti-Xa, antifactor Xa; UFH, unfractionated heparin; ECMO, extracorporeal membrane oxygenation.

^a^Median (IQR).

^b^Mean (SD).

^c^Chi-square tests were used to compare between various groups. For post-hoc testing pairwise chi-square tests, with the Bonferroni correction (*p* < 0.0167), were performed.

^d^Kruskal Wallis tests were used to compare between various groups. For post-hoc testing pairwise Mann Whitney tests, with the Bonferroni correction (*p* < 0.0167), were performed.

^e^ANOVA-F tests were used to compare between various groups. For post-hoc testing the Tukey HSD test was performed.

Table 4 presents exposure to blood products and anti-hemostatic agents. In both the ACT- and aPTT-guided group all patients (100%) received an RBC transfusion, while in the anti-Xa-guided group 91.7% of patients received a RBC transfusion (*p* = 0.049). The number of patients receiving platelets transfusion was similar over all three groups (*p* = 0.096), however the anti-Xa-guided group received a higher dose (*p* < 0.001). PCC was administered in 10% of ACT-guided patients, 5% of aPTT-guided patients and 25% of anti-Xa-guided patients (*p* = 0.021), post-hoc analysis identified a significant difference between aPTT- and anti-Xa-guided titration. Transfusions of other blood products did not differ between groups. Finally, tranexamic acid and protamine were significantly more often used in the ACT-guided group (respectively *p* = 0.001; *p* = 0.003).

**Table 4.** Exposure to blood products and anticoagulant medication.

	ACT (n = 30)	aPTT (n = 40)	anti-Xa (n = 48)	*p*
Median lowest Hb (g/dL) (n = 116)^a^	7.0 (6.7-7.2)^c^	6.6 (6.5-6.8)^cb^	6.6 (6.1-7.3)^c^	*0.006*
RBCs, no. (%)	30 (100)^d^	40 (100)^d^	44 (91.7)^d^	*0.049*
mL/kg d^-1^ (n = 114)^b^	31.1 (17.1–51.5)	17.4 (9.4–36.7)	22.2 (7.5–40.5)	*0.054*
Platelets, no. (%)	30 (100)	36 (90.0)	41 (85.4)	*0.096*
mL/kg d^-1^ (n = 107)^b^	3.5 (2.3–4.8)^c^	5.3 (2.6–8.4)^c^	10.5 (5.8–18.0)^c^	*<0.001*
Plasma^a^, no. (%)	29 (96.7)	37 (92.5)	43 (89.6)	*0.518*
mL/kg d^-1^ (n = 109)^b^	14.6 (6.1–33.5)^c^	8.4 (3.2–17.7)^c^	15.2 (7.3–25.0)^c^	*0.041*
PCC, no. (%)	3 (10.0)^d^	2 (5.0)^d^	12 (25.0)^d^	*0.021*
Fibrinogen, no. (%)	17 (56.7)	21 (52.5)	19 (39.6)	*0.275*
mg/kg d^-1^ (n = 57)^b^	36.2 (12.8–73.6)	27.9 (12.7–39.6)	23.8 (12.4–83.1)	*0.558*
Aspirin, no. (%)	1 (3.3)	6 (15.0)	6 (12.5)	*0.278*
Clopidogrel, no. (%)	0	2 (5.0)	0	*0.138*
Tranexamic acid (<24h-run), no. (%)	15 (50.0)^d^	8 (20.0)^d^	6 (12.5)^d^	*0.001*
Protamine (<8h-run), no. (%)	13 (43.3)^d^	6 (15.0)^d^	6 (12.5)^d^	*0.003*
Activated factor VII, no. (%)	3 (10.0)^d^	0^d^	0^d^	*0.011*

ACT: activated clotting time; aPTT: activated partial thromboplastin time; anti-Xa: antifactor Xa; RBC: red blood cell; PCC: prothrombin complex concentrates.

^a^Plasma consisted out of fresh frozen plasma and pooled plasma.

^b^Median (IQR).

^c^Kruskal Wallis tests were used to compare between various groups. For post-hoc testing pairwise Mann Whitney tests, with the Bonferroni correction (*p* < 0.0167), were performed.

^d^Chi-square tests were used to compare between various groups. For post-hoc testing pairwise chi-square tests, with the Bonferroni correction (*p* < 0.0167), were performed.

### Sensitivity analysis

Detailed tables of the secondary analysis are displayed in Supplement 2. Fourteen patients that were managed by multiple titration methods were excluded, leaving 104 runs of which 26 ACT-guided, 30 aPTT-guided, and 48 anti-Xa-guided (Supplemental Figure 1). Patients’ demographics were similarly distributed. Overall, similar results were seen between both analyses. There was no difference in complications between groups. Notably, the analysis newly identified the aPTT-guided group to have a significantly longer duration of intubation compared to anti-Xa-guided titration (ACT 14 days vs aPTT 33 days vs anti-Xa 17 days, *p* = 0.008). Also, a bolus was now significantly less often given in the aPTT-guided group compared to both groups (*p* = 0.003). The delay in administration of continuous heparin was no longer significant (*p* = 0.117). In contrast to the primary analysis, the median lowest hemoglobin level was no longer different between groups (*p* = 0.065). Also, no difference was seen in the number of RBC transfusions given (*p* = 0.088), and in the number of PCC transfusions given (*p* = 0.077). Anticoagulant medication was similarly distributed as in the primary analysis.

## Discussion

In this study, we observed no statistically significant differences between the different UFH titration strategies used over time and the occurrence of hemorrhagic complications. Secondary outcomes, including thrombosis, neurological complications and, survival were also not significantly different. Although no statistical difference was found, a trend towards increased hemorrhagic and decreased thrombotic complications was observed in the anti-Xa-guided group. However, this trend was not associated with an increased need for RBC transfusions, prolonged ECMO duration, prolonged hospitalization, or mortality. Interestingly, a significantly higher daily cumulative UFH dose was given in the anti-Xa-guided group. The most likely explanation for the increased UFH dose observed in the anti-Xa group is that the anti-Xa only measures UFH activity in contrast to the ACT and aPTT who are also prolonged in cases of coagulopathy or factor deficiency due to consumption. Both are frequent in children on ECMO. This can explain why UFH dose was lower in the ACT- and aPTT-group to achieve predetermined targets. A rise in UFH dose might also be explained by the presence of free hemoglobulin and bilirubin levels, that can falsely lower the anti-Xa level.^9^ However, in our population, most of the patients had only slightly elevated free hemoglobulin or bilirubin, although these findings were not included in the presented data.

Currently there is no consensus on the best titration method for UFH in pediatric ECMO patients and results of different studies are conflicting. A systematic review and meta-analysis that studied the correlation of the different laboratory results, including ACT, aPTT, anti-Xa, and thromboelastogram (TEG-R), to the UFH dose in children concluded that the anti-Xa had the best correlation with the heparin dose.^18^ The authors also studied whether the results of any of the laboratory test had an association with bleeding, thrombosis, or mortality, but found no association. These results are in line with our own results, showing no difference in complications or mortality between the three titration strategies used. Another systematic review and meta-analysis compared anti-Xa-based to time-based titration methods, including ACT, aPTT, ROTEM^®^ (Rotational Elastometry), and TEG-R, in patients receiving ECMO. The subgroup analysis in both neonates and children found the anti-Xa-guided titration associated with significantly less thrombotic events and a decreased mortality.^19^ Although not significant in our study, a trend towards decreased thrombosis was seen with no difference in mortality. These differences might be explained by the small sample size of our study. Nevertheless, both meta-analyses consisted of few studies with only a few high-quality studies, and a wide heterogeneity between them, therefore these results could be viewed as hypothesis-generating only. In a recent study not included in the previous meta-analyses, Rama and colleagues find also a non-significant trend towards increased hemorrhagic complications and no difference in thrombotic complications in anti-Xa-guided titration compared to ACT-guided titration.^20^ Coagulation and anti-coagulation is complex in children on ECMO. Besides the administration of anticoagulation, underlying clinical condition, cannulation strategy, circuit, coating, ECMO flow, blood products, hemostatic and anti-thrombotic agents, all impact coagulation. A broader evaluation than only heparin activity is needed to decrease hemorrhagic and thrombotic complications. A combination of multiple anticoagulation tests may be superior to a single test during ECMO.^19^

## Limitations

The retrospective character of the study increased the risk of unmeasured bias. Increasing group sizes over time may affect evolving clinical practice, introducing potential temporal bias. Also, due to the small sample size, our study may lack the power to detect a significant difference. Furthermore, due to the mono-centric character of the study and an overrepresentation of cardiac ECMOs in our population, the results should be interpreted cautiously and not generalized to other centers. In addition, the complications might have been underreported due to a few patients being discharged before the 14-days follow-up period. However, this group was small (n = 6) as the median hospital LOS ranged between 32 and 56 days in our population. Since these complications were considered “severe,” we would expect that they would have required readmission and therefore been captured. Finally, potential confounders may also exist given the large study time frame and rapidly evolving nature of technology and management in our patient group. Over the study period, there were no major changes in ECMO circuit and cannula, nor in institutional ECMO protocols regarding transfusion thresholds, indications, or dosing of haemostatic and antifibrinolytic agents. In this study different titration testing methods were compared but no changes in testing methods occurred over time. In our study, 14 patients were treated with multiple titration methods during transition periods. To assess the risk of bias, a sensitivity analysis was performed showing no difference in results.

## Conclusion

In conclusion, our study demonstrated that ACT-, aPTT, and anti-Xa-guided anticoagulation management used to manage heparin-dosing in children on ECMO showed no statistical differences in any complication and mortality. Importantly, a significant higher unfractionated heparin dose was given in the anti-Xa-guided group, which was associated with a non-significant rise in hemorrhagic complications and a non-significant decrease in thrombotic complications. These findings highlight the complexity of anticoagulation management in pediatric ECMO patients. A combination of tests might be superior to one method solely. Further studies should focus on a combinations of tests and their effects on complications to determine the best approach.

## Supplemental material


Supplemental material - Protocols for anticoagulation management in pediatric extracorporeal membrane oxygenation: A comparative retrospective study
Supplemental material for Protocols for anticoagulation management in pediatric extracorporeal membrane oxygenation: A comparative retrospective study by ML Jansen, NC Boom, N Schalij, PP Roeleveld, E Van Es, O Karam and A Willems in Perfusion

Supplemental material - Protocols for anticoagulation management in pediatric extracorporeal membrane oxygenation: A comparative retrospective study
Supplemental material for Protocols for anticoagulation management in pediatric extracorporeal membrane oxygenation: A comparative retrospective study by ML Jansen, NC Boom, N Schalij, PP Roeleveld, E Van Es, O Karam and A Willems in Perfusion

## Data Availability

The datasets used and analysed during the current study are available from the corresponding author on reasonable request.*
