# Supplementary material for: Protocols for anticoagulation management in pediatric extracorporeal membrane oxygenation: A comparative retrospective study
Source: Perfusion. 2026 Jan 21;41(7):1251–9. doi: 10.1177/02676591261416084 (PMC13624337; doi:10.1177/02676591261416084)
Supplement: Supplementary file 1 [file sj-pdf-1-prf-10.1177_02676591261416084.pdf]

Supplementary material to

Protocols for anticoagulation management in pediatric Extracorporeal Membrane Oxygenation: a comparative retrospective study

Supplementary material 1: definition of complications

Hemorrhagic complications:

- Hemorrhagic complications requiring packed red blood cell or whole blood (PRBC) transfusion (>20mL/kg d^{-1} of PRBCS or >3U PRBCs/calendar day in neonates and pediatrics and >3U PRBCS/calendar day in adults) or any other intervention such as surgical or endoscopic intervention. Complications are those associated with the ECLS run or as a consequence of the ECLS run.
- Peripheral cannulation site bleeding: In case there is bleeding from a peripheral cannulation site such as the neck, groin, or axilla. Peripheral cannulation site bleeding requiring PRBC transfusion (>20mL/kg d^{-1} of PRBCS or >3U PRBCs/day in neonates and pediatrics or >3U PRBCS/day in adults) and/or, surgical intervention (includes intravascular hemostatic agent deployment). A reperfusion cannula is a type of peripheral cannulation site.
- Mediastinal cannulation site bleeding: Mediastinal cannulations are also referred to as central cannulations and are placed via their mediastinum. Mediastinal cannulation site bleeding requiring PRBC transfusion (>20mL/kg d^{-1} of PRBCS or >3U PRBCs/day in neonates and pediatrics or >3U PRBCS/day in adults, and/or surgical intervention.
- Surgical site bleeding: In case of bleeding from a surgical site other than mediastinal or peripheral cannulation site. Requiring PRBC transfusion (>20mL/kg d^{-1} of PRBCS or >3U PRBCs/day in neonates and pediatrics or >3U PRBCS/day in adults), and/or surgical intervention.
- GI-hemorrhage: In case of upper or lower GI hemorrhage requiring PRBC transfusion (>20mL/kg d^{-1} of PRBCS or >3U PRBCs/day in neonates and pediatrics or >3U PRBCS/day in adults), and/or, endoscopic intervention, and/or hemostatic agent deployment.

- Pulmonary hemorrhage: In case of pulmonary hemorrhage requiring pRBC transfusion (>20mL/kg d^{-1} of PRBCS or >3U PRBCs/day in neonates and pediatrics and >3U PRBCS/day in adults).
- Intra cranial bleeding: In case of intraventricular and intra/extra parenchymal CNS hemorrhage. Intraventricular hemorrhage defined as>= grade 2 IVH on US, CT or MRI. Intra/extra parenchymal hemorrhage may be intraparenchymal, subdural or subarachnoid, confirmed by US, CT, or MRI.

Thrombotic complications:

- Inpatient thrombosis: clots found in major vessels through imaging and also strokes.
- Circuit change: entire circuit (with exception of cannulas) changed due to clot formation or mechanical failure
- Component change: circuit components (e.g. cannulas, oxygenator, tubing, pump) requiring change due to clot formation or mechanical failure of the component or equipment.
- Intracardiac thrombosis
- Limb ischemia
- Pulmonary embolism

Neurological complications:

- Seizure either confirmed clinically or with electroencephalogram.
- CNS diffuse ischemia: CT or MRI demonstrating diffuse ischemic changes.
- CNS infarction: CT, US, or MRI demonstrating localized ischemic change.
- Intraventricular CNS hemorrhage: >= grade 2 IVH on US, CT, or MRI.
- Intra/extra parenchymal CNS hemorrhage: may be intraparenchymal, subdural or subarachnoid, confirmed by US, CT, or MRI.

Infectious: positive cultures from blood, bone, cerebrospinal fluid, peritoneal fluid, pleural fluid, respiratory tract, skin/soft tissue, stool, urine, would (surgical or traumatic) for bacteria, fungi, viruses, prions and protozoa during PICU stay.

Renal replacement therapy: Peritoneal Dialysis (PD), Continuous Venovenous Hemodiafiltration (CVVHD), Continuous Venovenous Hemofiltration (CVVHF) or Continuous Venovenous Hemodiafiltration (CVVHDF) or Hemodialysis (HD) based on the patient's ultimate mode of therapy.

Necrotic enterocolitis: stage 2 or higher of the staging of Bell.
