# Supplementary material for: Protocols for anticoagulation management in pediatric extracorporeal membrane oxygenation: A comparative retrospective study
Source: Perfusion. 2026 Jan 21;41(7):1251–9. doi: 10.1177/02676591261416084 (PMC13624337; doi:10.1177/02676591261416084)
Supplement: Supplementary file 2 [file sj-pdf-2-prf-10.1177_02676591261416084.pdf]

Supplementary material to

Protocols for anticoagulation management in pediatric Extracorporeal Membrane Oxygenation: a comparative retrospective study

Supplementary material 2: Tables and figures of the sensitivity analysis

Contents

Supplementary figure 1

Supplementary figure 2

Supplementary table 1

Supplementary table 2

Supplementary table 3

Supplementary table 4

Supplementary figure 1: Flow diagram, sensitivity analysis

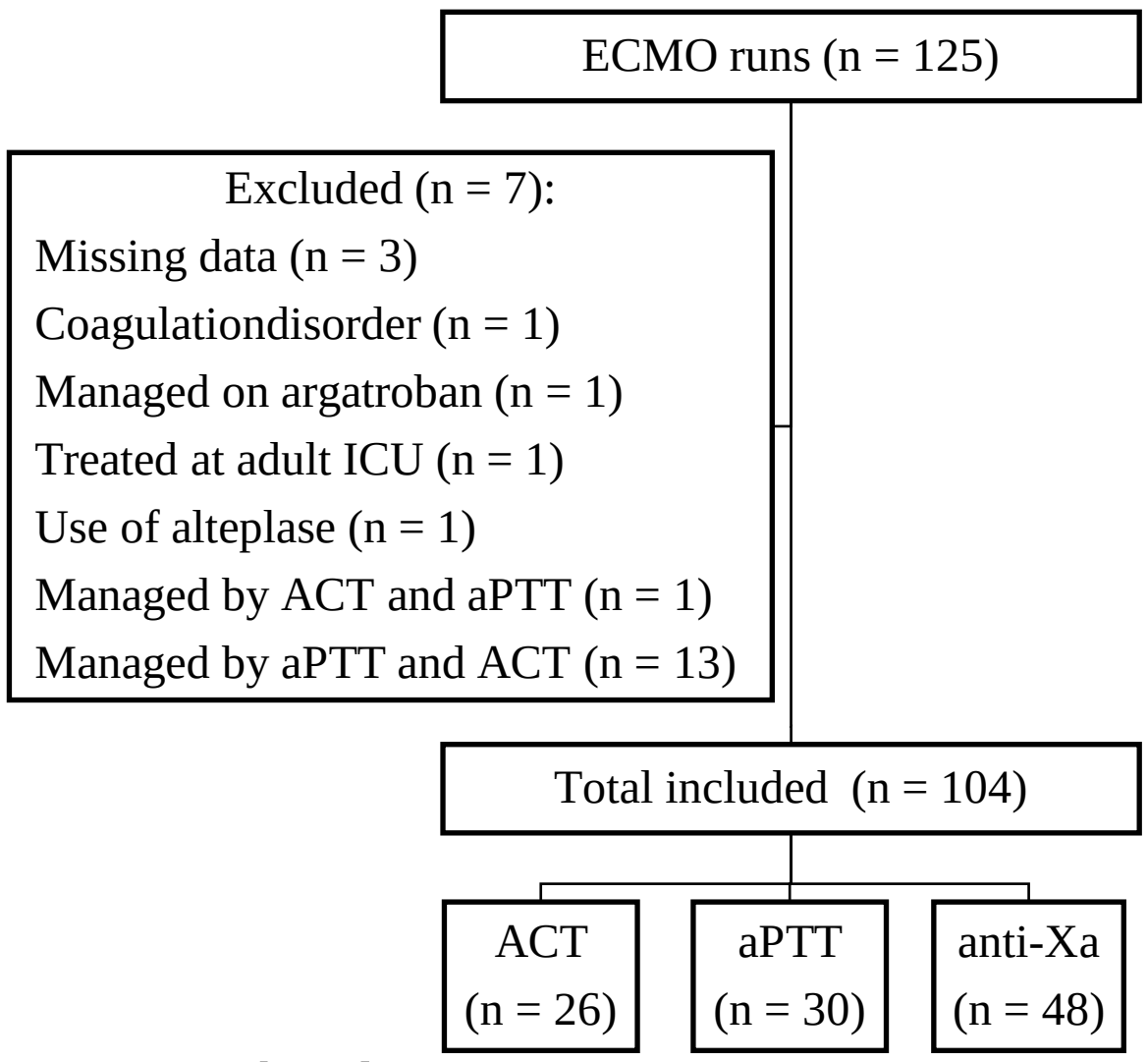

Figure 1. Flow diagram
ECMO, extracorporeal membrane oxygenation; ICU, intensive care unit; ACT, activated clotting time; aPTT, activated partial thromboplastin time; anti-Xa, antifactor Xa.

Supplementary figure 2: Kaplan-Meier, sensitivity analysis

[Insert: Figure4_KaplanMeier_sensitivity_analysis]

Figure 2. Kaplan-Meier analysis of 30-day survival after decannulation from extracorporeal membrane oxygenation support, sensitivity analysis. Log rank test (p = 0.558).

Supplementary table 1: Baseline characteristics, sensitivity analysis

	Total (n = 104)	ACT (n = 26)	aPTT (n = 30)	anti-Xa (n = 48)	*p*
Sex, males, no. (%)	45 (43.3)	13 (50.0)	9 (30.0)	23 (47.9)	*0.217*
Age (days) [1]	67.2 (10.2 – 306.9)	135.8 (18.5-323.3)	76.9 (19.7-337.1)	32.4 (2.3-125.8)	*0.120*
Weight (kg) [1]	4.2 (3.5-7.0)	6.0 (3.4-7.8)	4.5 (3.8-6.5)	4.0 (3.0-6.8)	*0.339*
Veno-arterial, no. (%)	101 (97.1)	26 (100)	30 (100)	45 (93.8)	*0.165*
Central cannulation (n = 101), no. (%)	57 (54.8)	17 (70.8)	13 (44.8)	27 (56.3)	*0.164*
Disorder, no. (%)					*0.056*
Chromosomal	13 (12.5)	7 (26.9)	2 (6.7)	4 (8.3)	
Infectious	5 (4.8)	3 (11.5)	1 (3.3)	1 (2.1)	
Congenital cardiac	83 (79.8)	23 (88.5)	29 (96.7)	31 (64.6)	
Acquired cardiac	11 (10.6)	1 (3.8)	5 (16.7)	5 (10.4)	
Pulmonary hypertension	25 (24.0)	4 (15.4)	7 (23.3)	14 (29.2)	
Congenital lung	12 (11.5)	0	3 (10.0)	9 (18.8)	
Acquired lung	6 (6.7)	3 (11.5)	2 (6.7)	2 (4.2)	
Indication ECMO, no. (%)					*0.136*
Cardiac	88 (84.6)	23 (88.5)	26 (86.7)	39 (81.3)	
Respiratory	26 (25.0)	5 (19.2)	8 (26.7)	13 (27.1)	
ECPR	33 (31.7)	5 (19.2)	10 (33.3)	18 (37.5)	
Sepsis	3 (2.9)	3 (11.5)	0	0	
Postoperative	50 (48.1)	16 (61.5)	14 (46.7)	20 (41.7)	

ACT: activated clotting time; aPTT: activated partial thromboplastin time; anti-Xa: antifactor Xa; ECMO: extracorporeal membrane oxygenator; ECPR: extracorporeal cardiopulmonary resuscitation;

[1] Median (IQR)

Supplementary table 2: Clinical course and complications, sensitivity analysis

	ACT (n = 26)	aPTT (n = 30)	anti-Xa (n = 48)	***p***
Duration of ECMO **(**days) [1]	4.0 (2.7-6.3)[a]	6.3 (3.2-8.8)[a]	3.5 (2.0-5.8)[a]	***0.026***
Duration intubation (days) [1]	14.0 (10.4-25.1)[ab]	33.3 (18.2-41.9)[a]	17.2 (7.9-28.1)[b]	***0.008***
Hospital LOS (days) [1]	32.0 (20.8-65.8)[a]	53.5 (36.0-86.0)[a]	31.5 (15.0-58.0)[a]	***0.036***
ICU LOS **(**days) [1]	21.0 (12.8- 40.8)[a]	45.0 (32.8-63.5)[a]	21.0 (8.3-52.0)[a]	***0.047***
Mortality on ECMO, no (%)	15 (31.1)	10 (33.3)	6 (23.1)	*0.674*
Hemorrhagic, no. (%)	11 (42.3)	18 (60.0)	29 (60.4)	*0.280*
Surgical site	6 (23.1)	9 (30.0)	17 (35.4)	*0.544*
Cannula-site	4 (15.4)	11 (36.7)	11 (22.9)	*0.168*
Gastro-intestinal	0	0	0	-
Intra-cranial	1 (3.8)	3 (10.0)	7 (14.6)	*0.355*
Pulmonary	2 (7.7)	4 (13.3)	2 (4.2)	*0.335*
Thrombotic, no. (%)	11 (42.3)	16 (53.3)	16 (33.3)	*0.217*
Patient thrombus	4 (15.4)	9 (30.0)	7 (14.6)	*0.206*
Circuit change	2 (7.7)	8 (26.7)	5 (10.4)	*0.073*
Component change	6 (23.1)	5 (16.7)	4 (8.3)	*0.208*
Intracardiac	2 (7.7)	2 (6.7)	2 (4.2)	*0.799*
Limb ischemia	1 (3.8)	2 (6.7)	0	*0.218*
Pulmonary embolism	1 (2.1)	0	1 (2.1)	*0.576*
Neurological, no. (%)	5 (19.2)	10 (33.3)	17 (35.4)	*0.332*
Seizure	1 (3.8)	7 (23.3)	8 (16.7)	*0.124*
CNS diffuse ischemia	1 (3.8)	3 (10.0)	6 (12.5)	*0.482*
CNS infarction	3 (11.5)	6 (20.0)	7 (14.6)	*0.667*
Intraventricular hemorrhage	0	1 (3.3)	3 (6.3)	*0.404*
Intra/extra parenchymal CNS hemorrhage	1 (3.8)	2 (6.7)	5 (10.4)	*0.580*
Infection, no. (%)	8 (30.8)	12 (40.0)	11 (22.9)	*0.274*
NEC, no. (%)	2 (7.7)	2 (6.7)	8 (16.7)	*0.315*
RRT, no. (%)	4 (15.4)	10 (33.3)	13 (27.1)	*0.302*

ACT: activated clotting time; aPTT: activated partial thromboplastin time; anti-Xa: antifactor Xa; ECMO: extracorporeal membrane oxygenator; LOS: length of stay; ICU: intensive care unit; CNS, central nervous system; NEC, necrotic enterocolitis; RRT, renal replacement therapy

[1] Median (IQR)

[a-c] Kruskal Wallis tests were used to compare between various groups. For post-hoc testing pairwise Mann Whitney tests, with the Bonferroni correction (p < 0.0167), were performed.

Supplementary table 3: Unfractionated heparin dosing, sensitivity analysis

	ACT (n = 26)	aPTT (n = 30)	anti-Xa (n = 48)	*p*
Bolus, no. (%)	24 (92.3)[a]	18 (60.0)[b]	42 (87.5)[a]	***0.003***
Bolus (IU/kg) (n = 84) [1]	300.0 (102.8-676.3)[d]	100.1 (28.8-158.3)[e]	142.7 (75.0-189.6)[de]	***0.008***
Timing continuous UFH				*0.910*
Before start ECMO, no. (%)	2 (7.7)	1 (3.3)	3 (6.3)	
Simultaneously, no. (%)	5 (19.2)	6 (20.0)	12 (25.0)	
After start ECMO, no. (%)	19 (73.1)	23 (76.7)	33 (68.8)	
Delay (hours) [1]	11.0 (3.0-15.0)	3.7 (1.5-5.0)	3.0 (1.9-5.8)	*0.117*
Total dosage (IU/kg d^{-1}) [1]	351.9 (231.3-561.1)[d]	555.8(415.2-690.5)[e]	838.6 (650.9-981.1)[f]	***0.000***
Mean rate (IU/kg h^{-1}) [2]	12.9 (±10.9)[g]	23.2 (±8.0)[h]	31.5 (±10.1)[i]	***0.000***
Minimal rate (IU/kg h^{-1}) [1]	5.9 (2.8-10.8)[d]	12.3 (7.9-20.0)[e]	23.4 (20.0-27.5)[f]	***0.000***
Maximal rate (IU/kg h^{-1}) [1]	23.8 (15.7-29.3)[d]	32.0 (24.9-41.3)[e]	41.0 (34.2-48.1)[f]	***0.000***

ACT: activated clotting time; aPTT: activated partial thromboplastin time; anti-Xa: antifactor Xa; UFH, unfractionated heparin; ECMO, extracorporeal membrane oxygenation;

[1] Median (IQR)

[2] Mean (SD)

[a-c] Chi-square tests were used to compare between various groups. For post-hoc testing pairwise chi-square tests , with the Bonferroni correction ($p < 0.0167$), were performed

[d-f] Kruskal Wallis tests were used to compare between various groups. For post-hoc testing pairwise Mann Whitney tests, with the Bonferroni correction ($p < 0.0167$), were performed.

[g-i] ANOVA-F tests were used to compare between various groups. For post-hoc testing the Tukey HSD test was performed.

	ACT (n = 26)	aPTT (n = 30)	anti-Xa (n = 48)	*p*
Median lowest Hb (g/dL) (n = 102) [1]	7.0 (6.6-7.2)	6.6 (6.5-6.8)	6.6 (6.1-7.3)	*0.065*
RBCs, no. (%)	26 (100)	30 (100)	44 (91.7)	*0.088*
mL/kg d^{-1} (n = 100) [1]	33.2 (17.9-59.1)[a]	17.4 (10.4- 33.2)[b]	22.2 (7.5-40.5)[ab]	***0.043***
Platelets, no. (%)	26 (100)	27 (90.0)	41 (85.4)	*0.127*
mL/kg d^{-1} (n = 94) [1]	3.5 (2.1-5.5)[a]	7.6 (3.8-11.2)[a]	10.5 (5.8-18.0)[b]	***0.000***
Plasma(n=)*, no. (%)	24 (92.3)	29 (96.7)	43 (89.6)	*0.521*
mL/kg d^{-1} (n = 96) [1]	19.4 (6.9-37.5)[a]	8.1 (3.2-18.6)[a]	15.2 (7.3-25.0)[a]	***0.023***
PCC, no. (%)	3 (11.5)	2 (6.7)	12 (25.0)	*0.077*
Fibrinogen, no. (%)	12 (46.2)	16 (53.3)	19 (39.6)	*0.491*
mg/kg d^{-1} (n = 47) [1]	34.9 (12.1-107.6)	22.8 (11.2-45.6)	23.8 (12.4-83.1)	*0.528*
Aspirin, no. (%)	0	4 (13.3)	6 (12.5)	*0.157*
Clopidogrel, no. (%)	0	1 (3.3)	0	*0.288*
Tranexamic acid (24h prior run), no. (%)	13 (50.0)[d]	5 (16.7)[e]	6 (12.5)[e]	***0.001***
Protamine (8h prior run), no. (%)	12 (46.2)[d]	4 (13.3)[e]	6 (12.5)[e]	***0.002***
Activated factor VII, no. (%)	3 (11.5)[d]	0[de]	0[e]	***0.010***

ACT: activated clotting time; aPTT: activated partial thromboplastin time; anti-Xa: antifactor Xa; RBC: red blood cell; PCC: prothrombin complex concentrates;

* plasma consisted out of fresh frozen plasma and omni plasma.

[1] Median (IQR)

[a-c] Kruskal Wallis tests were used to compare between various groups. For post-hoc testing pairwise Mann Whitney tests, with the Bonferroni correction ($p < 0.0167$), were performed.

[d-f] Chi-square tests were used to compare between various groups. For post-hoc testing pairwise chi-square tests, with the Bonferroni correction ($p < 0.0167$), were performed

Supplementary table 4: Exposure to blood products and anticoagulant medication, sensitivity analysis
